# Mapping the research landscape of microRNAs in pain: a comprehensive bibliometric analysis

**DOI:** 10.3389/fnmol.2024.1493822

**Published:** 2024-12-24

**Authors:** Huaiming Wang, Qin Li, Jiang Zou, Jinjun Shu, Aimin Zhang, Hongwei Zhang, Qi Zhao, Shunxin Liu, Chan Chen, Guo Chen

**Affiliations:** ^1^Department of Anesthesiology, West China Hospital, Sichuan University, Chengdu, China; ^2^Department of Anesthesiology, Sichuan Clinical Research Center for Cancer, Sichuan Cancer Hospital & Institute, Sichuan Cancer Center, Affiliated Cancer Hospital of University of Electronic Science and Technology of China, Chengdu, China; ^3^Sichuan Women’s and Children’s Hospital, Women’s and Children’s Hospital, Chengdu Medical College, Chengdu, China; ^4^Guangxi University of Chinese Medicine, Nanning, China; ^5^The Research Units of West China (2018RU012)-Chinese Academy of Medical Sciences, West China Hospital, Sichuan University, Chengdu, China

**Keywords:** bibliometric analysis, microRNA, pain, Web of Science, miRNA

## Abstract

**Background and objectives:**

MicroRNAs (miRNAs) have demonstrated significant potential in pain medicine research, including mechanisms, diagnosis, and therapy. However, no relative bibliometric analysis has been performed to summarize the progress in this area quantitatively.

**Methods:**

Literature was retrieved from the Web of Science Core Collection online database. A total of 1,295 papers were retrieved between January 1, 2000 and September 21, 2023 and underwent visualization and analysis using R software [Library [bibliometrix] and biblioshiny packages], VOSviewer (version 1.6.18), CiteSpace software (version 6.2.R4), and the bibliometrics website (http://bibliometric.com).

**Results:**

Publications in this field have increased annually since 2000, demonstrating growing research interest. China emerged as the most productive country, followed by the United States and Germany. Keyword analysis identified “expression,” “neuropathic pain,” and “microRNAs” as the most relevant keywords. Extensive collaboration among countries and institutions was also observed.

**Conclusion:**

The bibliometric analysis revealed a rapid growth of publications related to miRNAs and pain in the past 2 decades. Keywords analysis indicates that “expression,” “neuropathic pain,” and “microRNA” are the most frequently used words in this research field. However, more robust and globally recognized basic studies and clinical trials from prestigious journals are required.

## 1 Introduction

Pain is an aversive sensory and emotional experience typically caused by, or resembling, an actual or potential tissue injury (Raja et al., 2020). It often manifests as a comorbidity with various clinical complaints, including sensory discomfort, emotional disorders, cognitive impairment, and even social or family problems, causing significant distress to patients and their families (Raja et al., 2020; Zhang X. et al., 2023). Pain generation, progression, and management mechanisms have been extensively studied both macroscopically and microscopically.

MicroRNAs (miRNAs) are a class of small non-coding RNAs that can target numerous protein-coding genes and are involved in the evolutionary and pathological progression of animals and humans. By controlling post-transcriptional gene expression, miRNAs are involved in various diseases (Saliminejad et al., 2019). Many studies have revealed that miRNAs can modulate pain, with many miRNAs being upregulated or downregulated in response to tissue or nerve injury (Morchio et al., 2023; Tao et al., 2023; Vali et al., 2023). This modulation affects target miRNAs, either suppressing or promoting pain generation. These findings demonstrate that targeting miRNAs could be an essential pathway in pain pathophysiology and therapeutics (Zhang X. et al., 2023; Saliminejad et al., 2019; Lopez-Gonzalez et al., 2017; Vali et al., 2023; Sakai and Suzuki, 2015; Tao et al., 2018). Nevertheless, the articles or reviews rarely present an intuitive and visual mapping of the research trends and highlights in this specific field.

Visualized bibliometric analysis is a novel and efficient method for providing an understandable review of prominent publications over a specific period (Chen et al., 2014). A recently published bibliometric analysis of the global study trends on neuropathic pain and epigenetics focused on the extensive function of genetics. It revealed some information and frontiers in epigenetics and neuropathic pain, especially DNA methylation, circular RNA, acetylation, and long non-coding RNA. However, a minuscule portion was associated with miRNA and the retrieve keyword mainly centered on all types of neuropathic pain (Zhu et al., 2023). The literature on pain and miRNA has been rapidly increasing, but no systematic review of these publications has yet been conducted. Therefore, this study systematically reviewed publications to explore the development of this field, reviewed key publications, assessed current research focus, forecasted future trends, and provided an overview for researchers.

## 2 Materials and methods

### 2.1 Data retrieval strategy

The Web of Science (WOS) has been globally used for bibliometric analysis due to its high-quality literature (Zhiguo et al., 2023). We searched the WOS Core Collection (WOSCC) on September 21, 2023, for publications related to pain and miRNA reported between January 1, 2000, and September 21, 2023. The retrieval formula was as follows: Topic Subject (TS) = (“miRNA” or “microRNA” or “miRNAs” or “MicroRNA” or “RNA Micro”) and (“pain” or “ache”), with the language limited to English. All included articles featured titles, abstracts, and keywords related to pain and miRNA.

### 2.2 Data screening process

**Figure 1:**
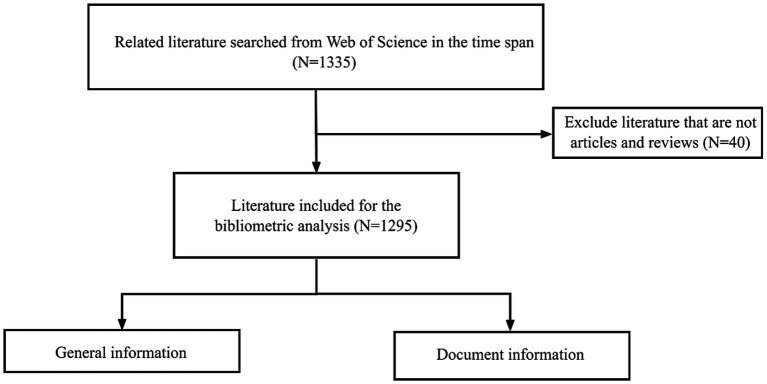
Process of article retrieval.

A total of 1,335 papers were retrieved. Two authors independently reviewed each paper to determine relevance and adherence to the inclusion criteria and exclusion criteria (listed below). If there was any uncertain paper after their evaluation, a third author was assigned to participate in a thorough discussion on that publication. If disagreement persisted, one of the corresponding authors reviewed the problematic publication to make the final decision. Lastly, 40 publications were excluded owing to the improper type of article. The final bibliometric analysis encompassed 1,295 articles. The literature retrieval and data screening process was illustrated in Figure 1 and presented as a flowchart.

### 2.3 Inclusion criteria

1. The study focused on pain and miRNA, with full text available.
2. The article type was either an article, review, or online publication.
3. The publication language was English.
4. The duration of the publication ranged from January 1, 2000, to September 21, 2023.
5. The literature was sourced from WOSCC.

### 2.4 Exclusion criteria

1. Topics not related to pain and miRNA.
2. Publication types including conference papers, abstracts, book chapters, theses, letters, corrections, withdrawn contributions, and reports.
3. Publications outside the defined time duration.

### 2.5 Data analysis

**Figure 2:**
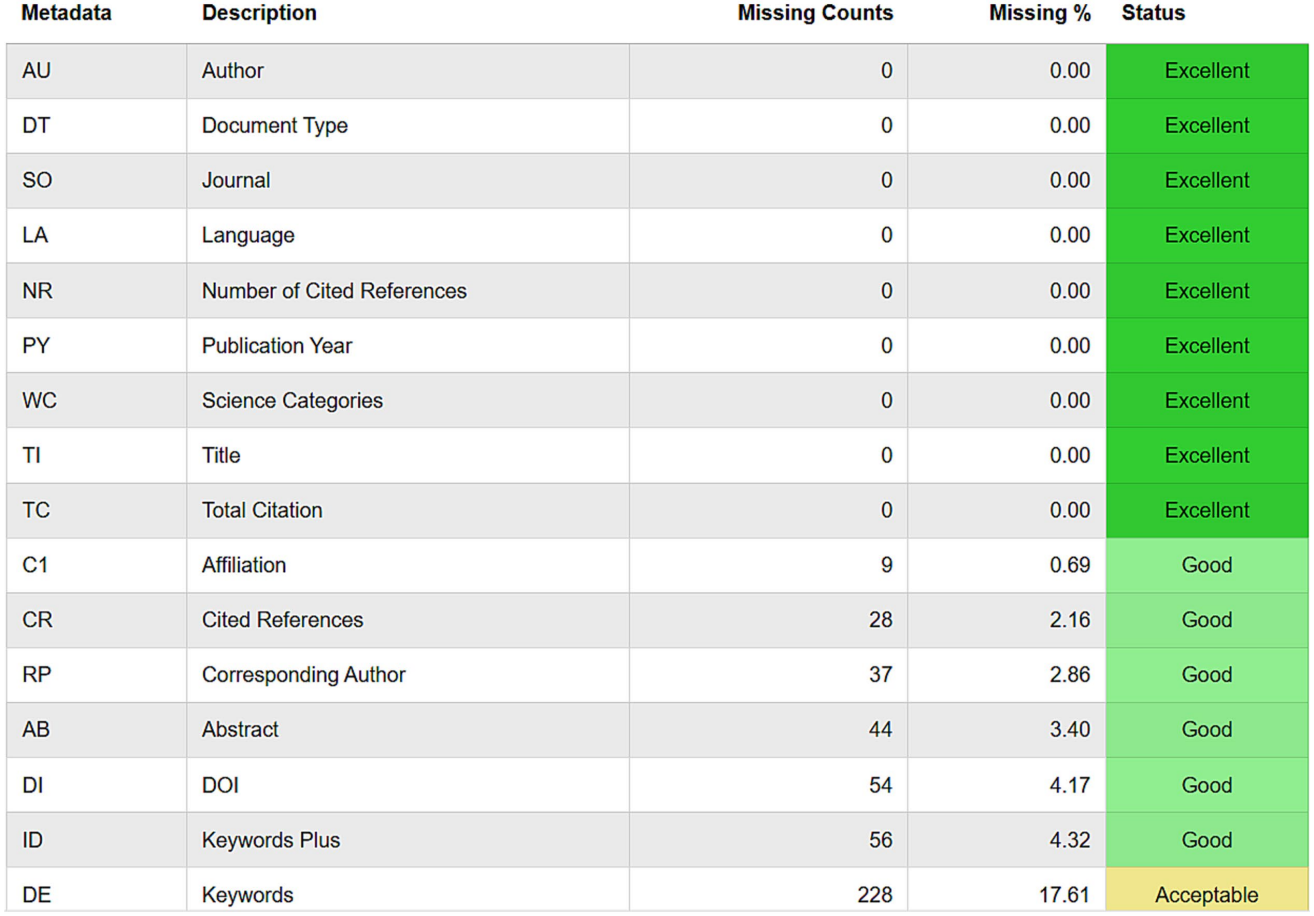
The completeness of bibliographic metadata.

To gain a comprehensive understanding of the current research on pain and miRNA, we used R software [library (bibliometrix) package and biblioshiny online tool] to visualize and analyze various aspects of the literature. First and foremost, the data quality was checked before the formal analysis by evaluating the completeness of bibliographic metadata (Figure 2). The following analysis included main information, annual scientific production, average citations per year, three-field plots, most relevant sources, authors, and affiliations, most locally cited sources, authors, documents, and references, affiliation production over time, corresponding author countries/regions, country scientific production, country production over time, most globally cited documents, most frequent words, tree map of words, word frequency over time, trending topics, clustering by coupling, co-occurrence network, thematic map, factorial analysis, co-citation network, collaboration network, and country collaboration maps.

Co-authorship, co-citation, co-occurrence, citation patterns, and bibliographic coupling were visually studied using VOSviewer (version 1.6.18) and CiteSpace software (version 6.2.R4). Burst keywords were visualized and analyzed using CiteSpace to identify trends in pain and miRNA research.

The Bibliometrics website^1^ was also used to analyze the annual publication output of the top 10 countries in this field and create a collaboration map among these countries.

Visualization maps are displayed as nodes and links. Nodes represented individual elements, such as keywords, countries, institutions, or authors, while linear links between nodes symbolized cooperation, co-citations, or occurrences among them. Nodes and links were color-coded to represent different years.

## 3 Results

### 3.1 General publication information and global production growth trend

**Figure 3:**
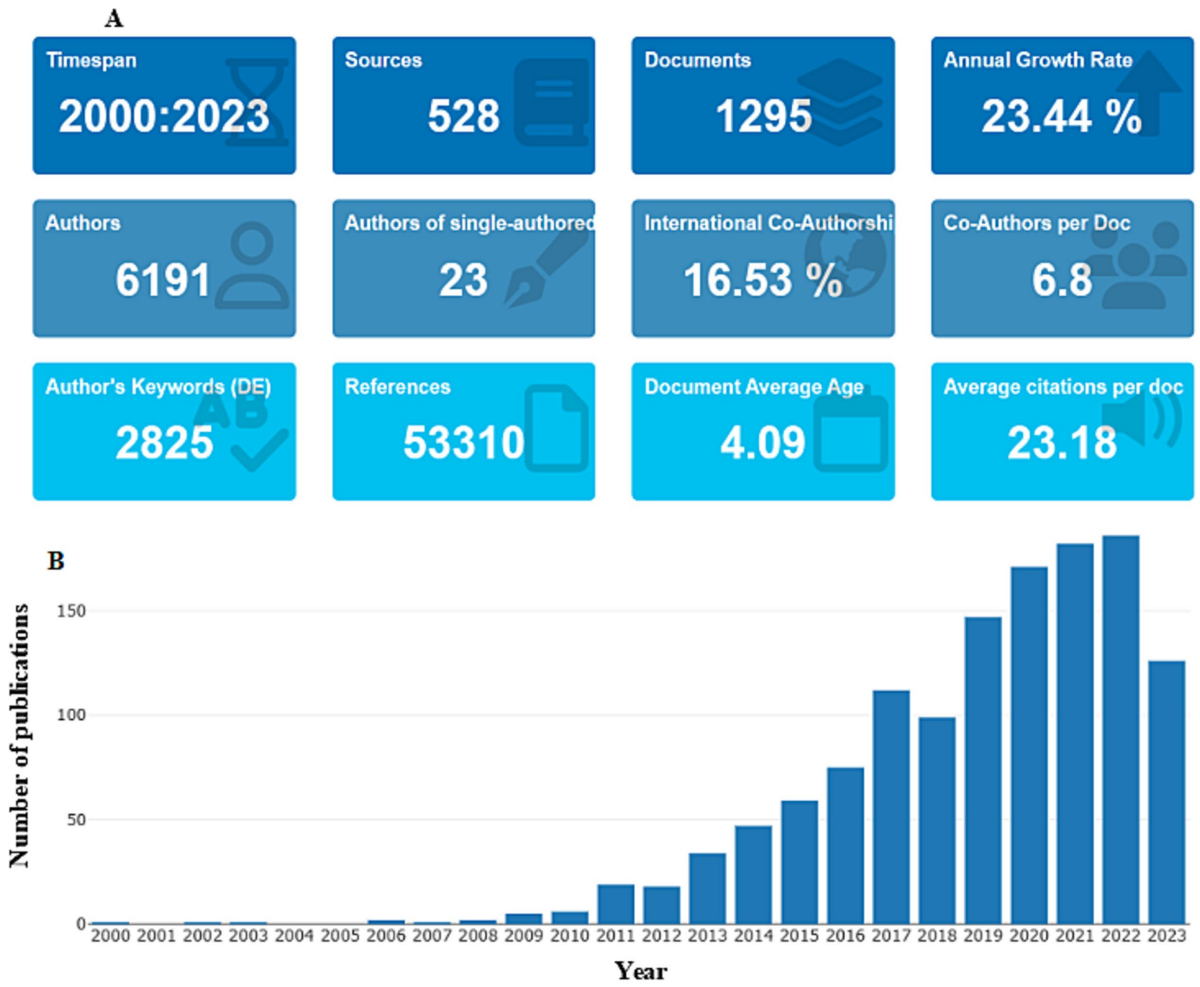
**(A)** General publication information. **(B)** Global production trend.

From January 1, 2000, to September 21, 2023, 6,191 authors from 52 countries contributed 1,295 publications across 528 international journals. The number of publications increased at an average annual rate of 23.44%, except for a decline in 2023 due to incomplete data retrieval for that year. Since 2010, there has been a significant increase in publications, indicating growing attention to research in pain and miRNAs and a positive outlook for advancements in pain medicine research (Figure 3).

### 3.2 Analysis of country/region distribution

**Figure 4:**
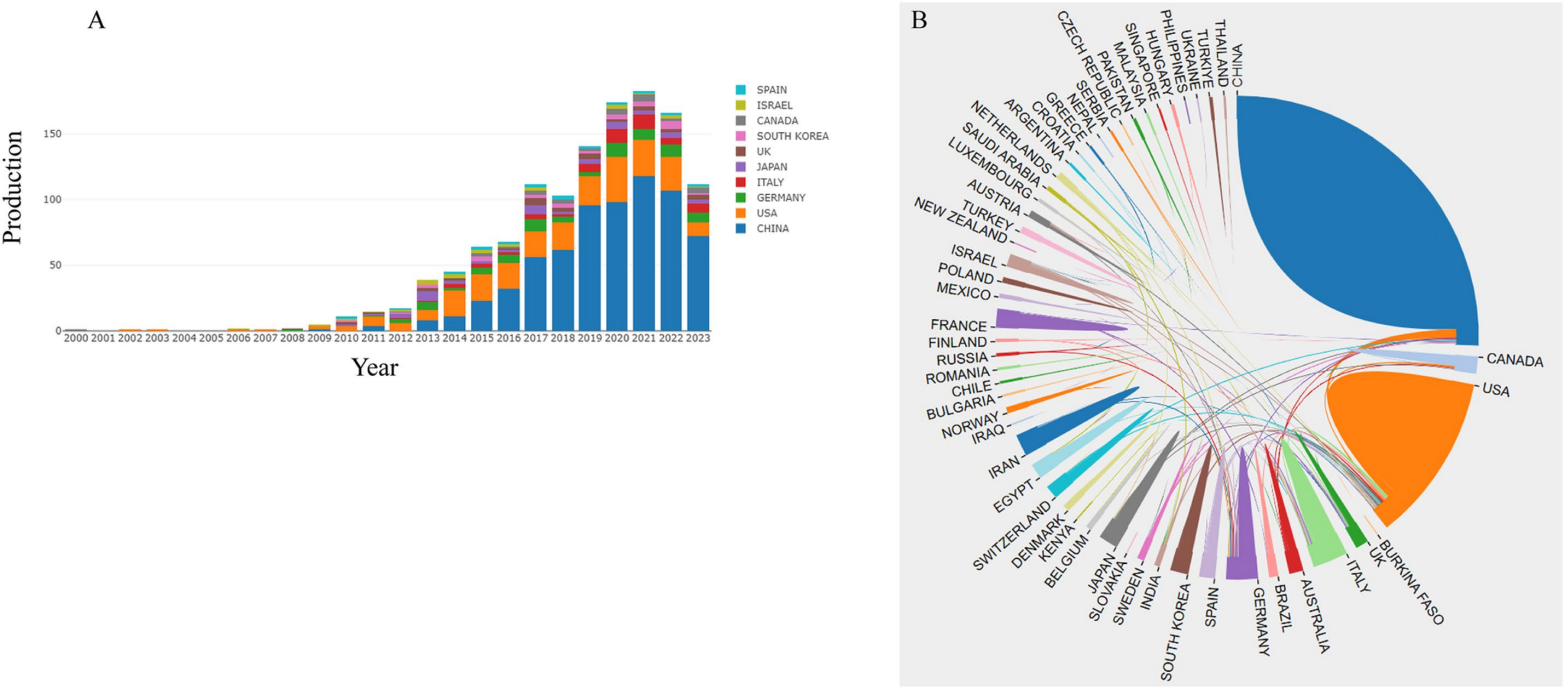
Distribution of countries by publication. **(A)** Annual publication of the top 10 countries; **(B)** The corporation map between the countries.

We investigated the distribution of publications across countries and discovered widespread interest in this research area. Authors from 52 different countries published papers on pain and miRNA, with Chinese authors dominating the field, contributing most of the papers, followed by researchers from the United States, Germany, Italy, Japan, the UK, South Korea, Canada, Israel, and Spain (Figure 4).

### 3.3 Keyword co-occurrence frequency and citation burst

Keyword co-occurrence frequency in the retrieved literature can provide insights into research interests, topics, and investigation trends in this scientific field. Using CiteSpace (version 6.2.R4), we generated keyword occurrence frequency statistics and visualized network mapping. We identified the top 10 frequent keywords: “expression” (*n* = 431), “neuropathic pain” (*n* = 311), “micro RNAs” (*n* = 142), “activation” (*n* = 121), “cells” (*n* = 120), “pain” (*n* = 114), “inflammation” (*n* = 112), “cancer” (*n* = 101), “gene expression” (*n* = 98), and “downregulation” (*n* = 95) (Table 1).

**Table 1:** Top 10 most frequently occurred keywords.

Rank	Keywords	Centrality	Count/frequency
1	Expression	0.1	431
2	Neuropathic pain	0.07	311
3	Micro RNAs	0.02	142
4	Activation	0.04	121
5	Cells	0.09	120
6	Pain	0.01	114
7	Inflammation	0.03	112
8	Cancer	0.02	101
9	Gene expression	0.13	98
10	Down regulation	0.05	95

**Figure 5:**
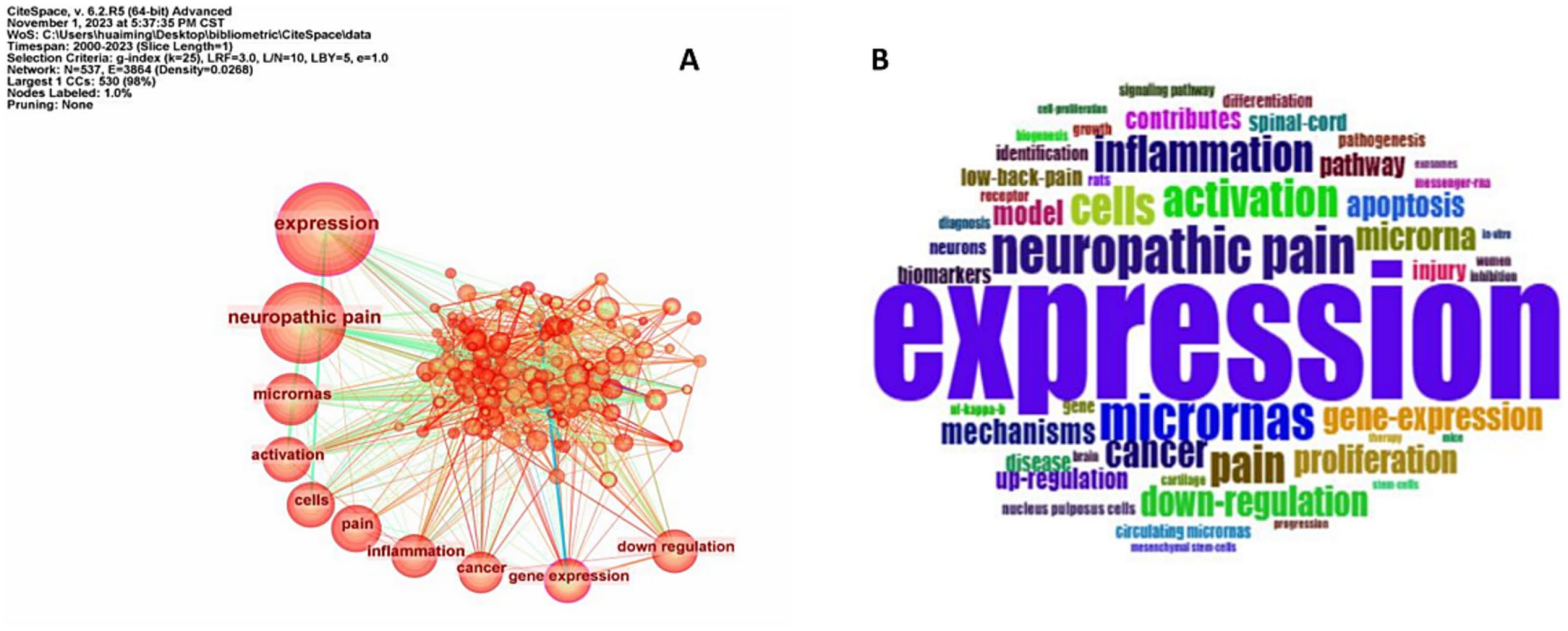
Analysis of **(A)** keyword co-occurrence and **(B)** keyword cloud in pain and micro RNA research.

The keyword network highlighted both the frequency of co-occurrence and relationships between keywords. “Expression,” “neuropathic pain,” and “microRNAs” were the top three frequent keywords highlighting that neuropathic pain, a persistent and unyielding pain is a primary focus in research examining the function of miRNAs in pain modulation and treatment (Figure 5).

**Figure 6:**
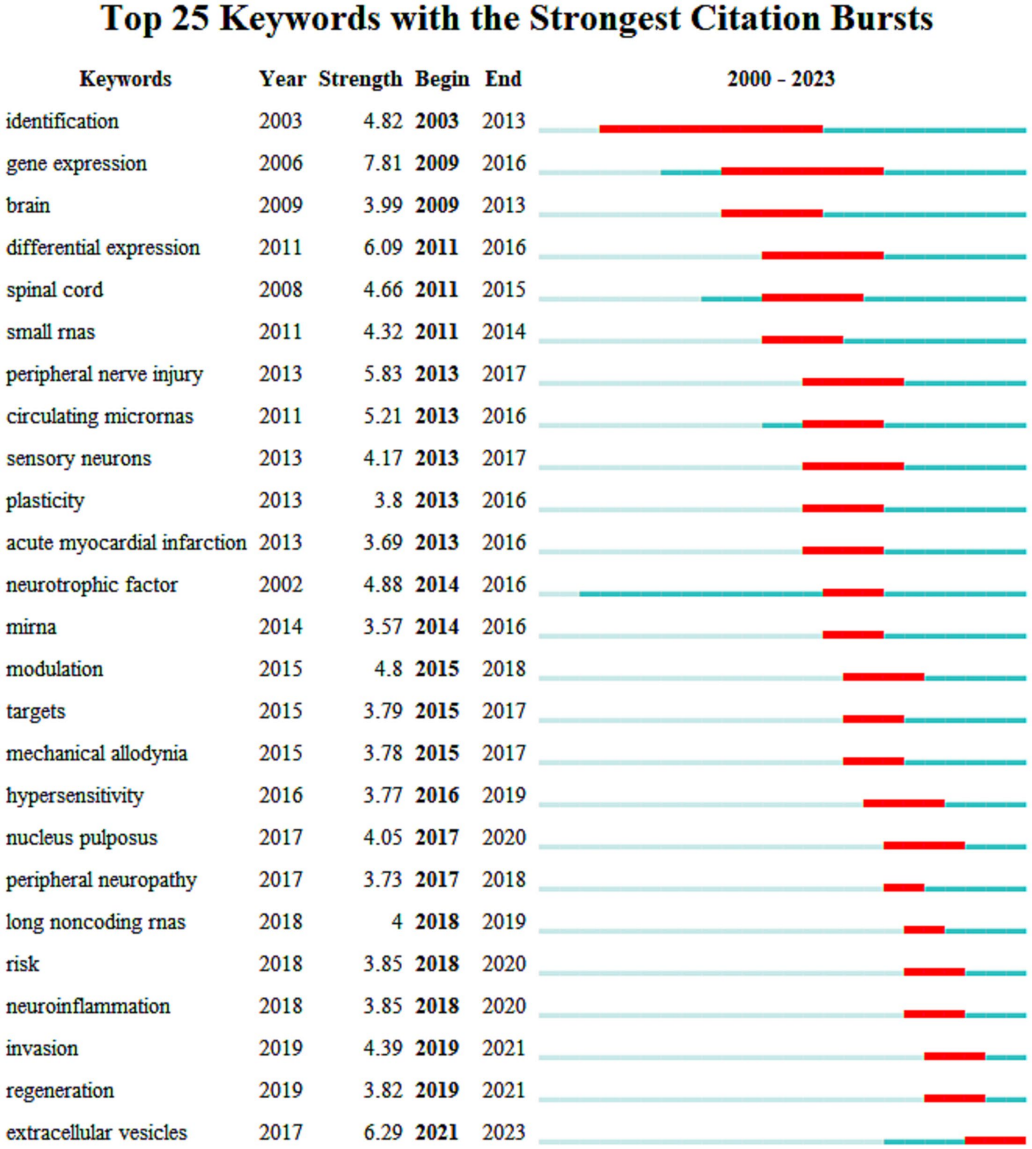
The top 25 keywords with the strongest citations burst in pain and miRNAs research.

Citation burst analysis of keywords revealed dynamic shifts in research topics over time. The top 25 keywords with the strongest citation bursts in pain and miRNA research are presented in Figure 6. Initially, gene identification and differential expression in pain-related diseases were the primary focus, particularly targeting the brain and spinal cord. However, in recent years, the focus has shifted to emerging topics such as small RNAs, long non-coding RNAs, and extracellular vesicles, reflecting evolving global research interests in the field of pain and miRNA.

### 3.4 Most productive and cited journal analysis

An online bibliometric analysis (see text footnote 1) identified 528 journals that published papers on pain and miRNAs during the analysis period. The most active journal was PLoS One (*n* = 29), followed by International Journal of Molecular Sciences (*n* = 27), Experimental and Therapeutic Medicine (*n* = 25), Journal of Cellular Biochemistry (*n* = 24), Molecular Pain (*n* = 22), Frontiers in Molecular Neuroscience (*n* = 21), Scientific Reports (*n* = 19), Pain (*n* = 18), Molecular Medicine Reports (*n* = 18), and Molecular Neurobiology (*n* = 17). The impact factors of the top 10 journals ranged from 2.7 to 7.4, with JCR (Journal Citation Report) quartiles distributed from Q1 to Q4 (Table 2).

**Table 2:** Top 10 productive journals in the research of microRNAs and pain.

Rank	Journal	Counts	Citations	Citations per paper	Impact factors (2022)	JCI quartile (2022)
1	PLoS One	29	175	6.03	3.7	Q3
2	International Journal of Molecular Science	27	57	2.11	5.6	Q2
3	Experimental and Therapeutic Medicine	25	47	1.88	2.7	Q4
4	Journal of Cellular Biochemistry	24	133	5.54	4	Q2
5	Molecular Pain	22	274	12.45	3.3	Q3
6	Frontiers in Molecular Neuroscience	21	161	7.67	4.8	Q2
7	Scientific Reports	19	67	3.53	4.6	Q3
8	Pain	18	188	10.44	7.4	Q1
9	Molecular Medicine Reports	18	43	2.39	3.4	Q4
10	Molecular Neurobiology	17	91	5.35	5.1	Q2

**Figure 7:**
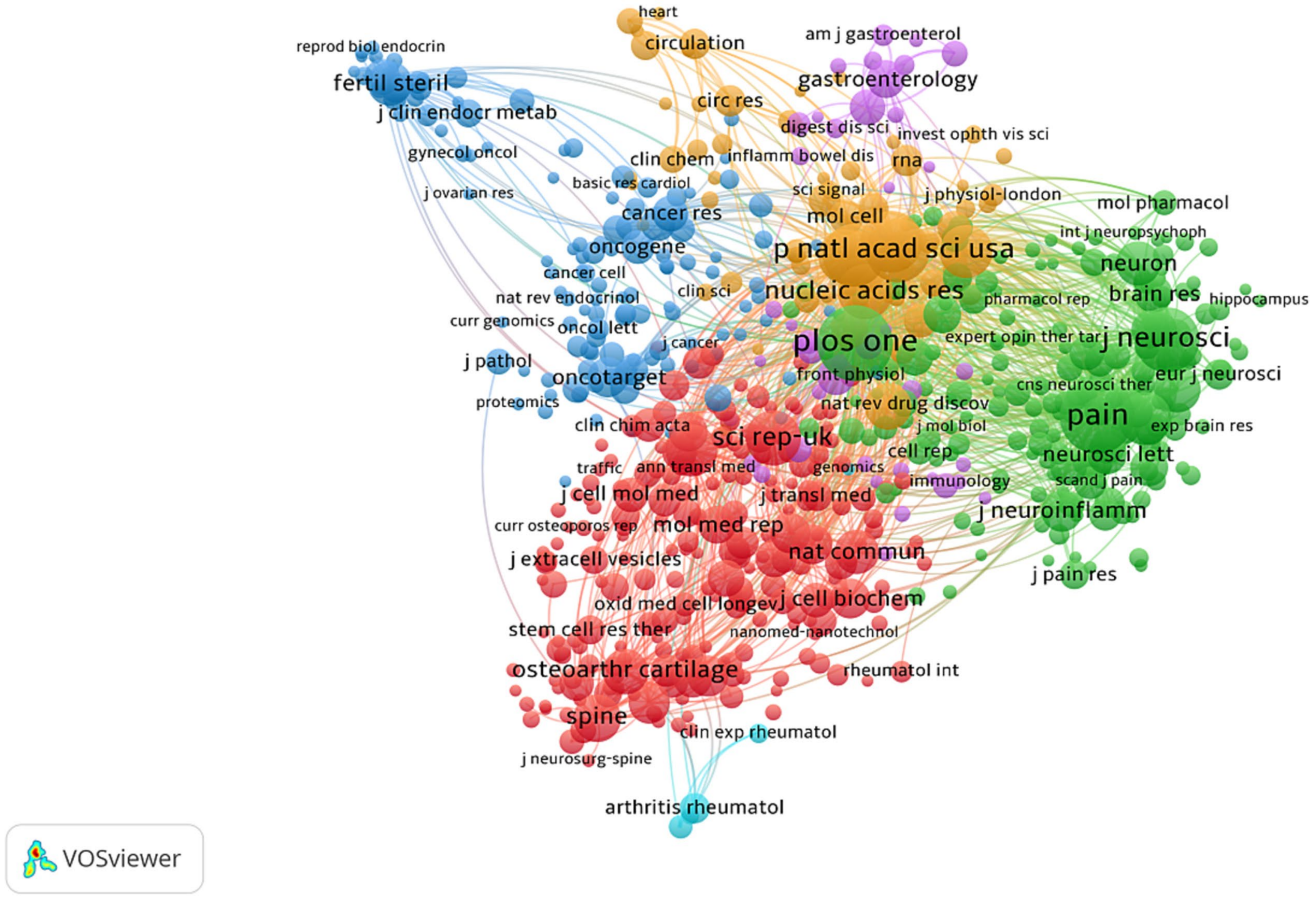
Network of co-citations between these journals. Diversified colored nodes represent different journals. The size of the node stands for the strength of the co-citations among journals.

Additionally, some articles were published in renowned global journals, including Science (*n* = 1) and Nature Communications (*n* = 3). The most cited journal was Molecular Pain (*n* = 274), with an average of 12.45 citations per paper (Table 3). The network of co-cited journals generated by VOSviewer (version 1.6.18) demonstrated strong communication and citation links among these journals (Figure 7).

**Table 3:** Top 10 most cited journals in the research of microRNAs and pain.

Rank	Journal	Citations	Citations per paper	Impact factors (2022)	JCI quartile (2022)
1	Molecular Pain	274	12.45	3.3	Q3
2	Pain	188	10.44	7.4	Q1
3	PLoS One	175	6.03	3.7	Q3
4	Frontiers in Molecular Neuroscience	161	7.67	4.8	Q2
5	Journal of Neuroinflammation	155	11.92	9.3	Q1
6	International Journal of Molecular Medicine	153	11.77	5.4	Q3
7	Biochemical and Biophysical Research Communications	147	11.31	3.1	Q4
8	Neurochemical Research	147	14.7	4.4	Q3
9	Journal of Cellular Biochemistry	133	5.54	4	Q2
10	Journal of Neuroscience	125	20.83	5.3	Q1

### 3.5 Analysis of author production, citation information

**Figure 8:**
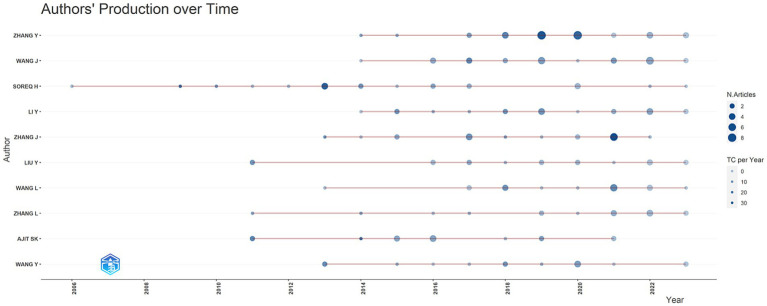
Production by authors over time. The size of the circle represents the number of articles.

We analyzed author production and impact on pain and miRNA research over time using R software (Library [Bibliometrix] and Biblioshiny package). Table 4 and Figure 8 summarize the output from the top 10 authors since 2000. Zhang Y. from the State Key Laboratory of NBC Protection for Civilians in Beijing, China, was the most productive researcher in this field, followed by Wang J. from China and Soreq H. from Israel. Of the top 10 most contributive authors, 80% were from China (Table 4), highlighting significant interest and contributions to studying the complex relationship between miRNA and pain.

**Table 4:** Authors’ accumulative total production from January 1st, 2000 to September 21st, 2023.

Rank	Authors	Countries	Articles
1	Zhang Y.	China	33
2	Wang J.	China	26
3	Soreq H.	Israel	21
4	Li Y.	China	20
5	Zhang J.	China	19
6	Liu Y.	China	17
6	Wang L.	China	17
7	Zhang L.	China	16
8	Ajit S. K.	USA	15
8	Wang Y.	China	15

**Figure 9:**
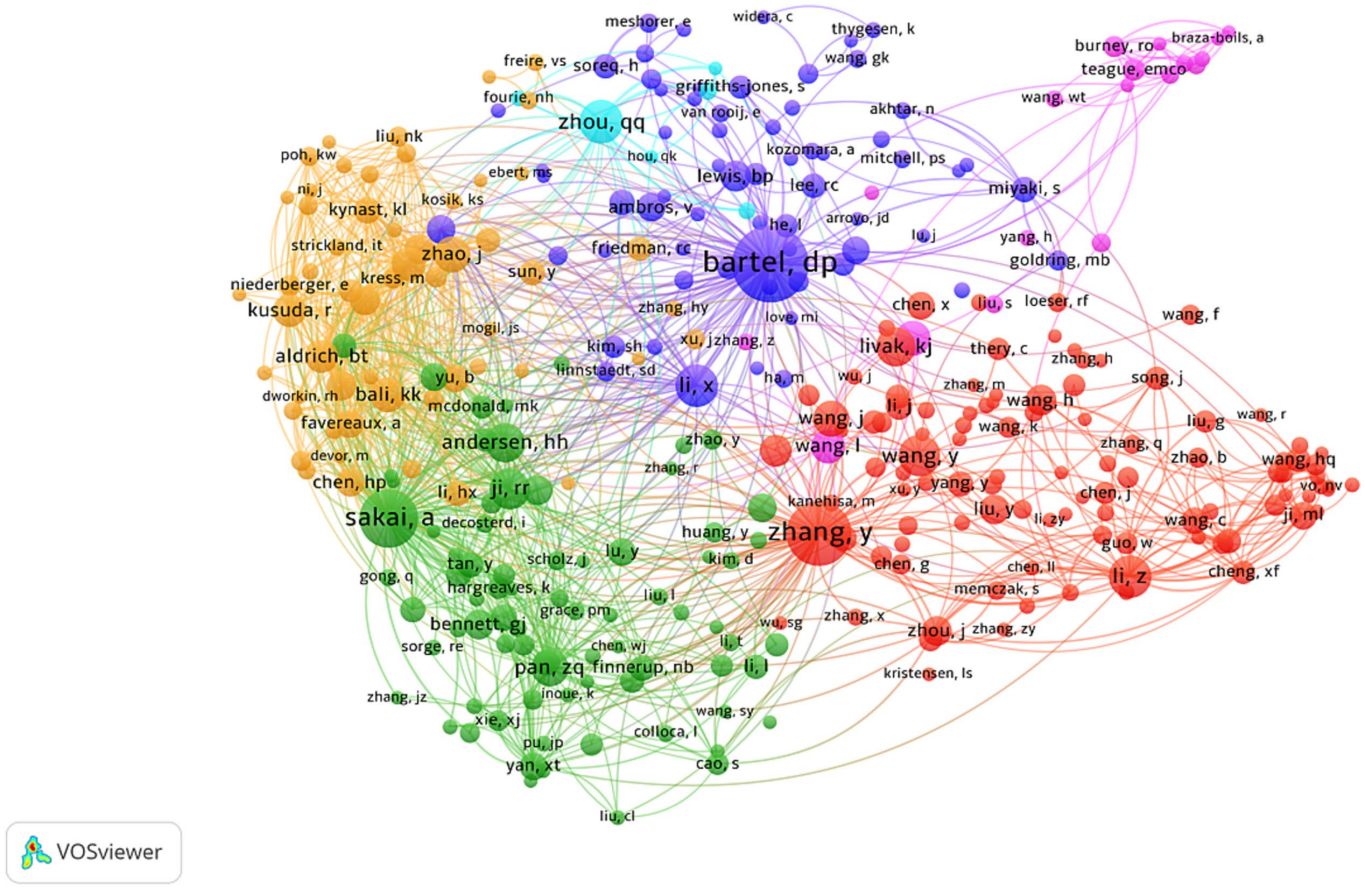
Network of co-cited authors. Nodes represent authors, with their size indicating the number of citations and curved lines representing co-citations between different authors.

The visualized network of co-cited authors by VOSviewer (version 1.6.18) revealed that Barel D. P., Zhang Y., and Sakai A. were the three most frequently cited researchers in this field, with active citation exchanges among them (Figure 9).

Besides, the citation information was analyzed using R software [Library [Bibliometrix] and Biblioshiny package]. The top 10 most-cited papers were listed in Table 5, in which the total citations (TC), TC per year, and normalized TC of each paper were presented as well. We found that the phase I study of liposomal miR-34a mimic for solid tumor therapy was most cited, followed by another research of miR-16-based mimic minicell intravenous infusion in human for recurrent malignant pleural mesothelioma and the study of MRX34 treatment for advanced solid tumor. Although, these miRNA therapy were related to cancer, however, the adverse effects included chronic pain commonly.

**Table 5:** List of the top 10 most-cited papers.

Rank	Paper	DOI	TC	TC per year	Normalized TC
1	Beg M. S. (2017). Invest. New Drug	10.1007/s10637-016-0407-y	534	66.75	12.73
2	van Zandwijk N. (2017). Lancet Oncol.	10.1016/S1470-2045(17)30621-6	394	49.25	9.39
3	Hong D. V. S. (2020). Br. J. Cancer	10.1038/s41416-020-0802-1	344	68.80	20.94
4	Shaked I. (2009). Immunity	10.1016/j.immuni.2009.09.019	338	21.13	2.35
5	Sommer C. (2018). Pain	10.1097/j.pain.0000000000001122	251	35.86	8.95
6	McDonald M. K. (2014). Pain	10.1016/j.pain.2014.04.029	227	20.64	4.31
7	Descalzi G. (2015). Trends Neurosci.	10.1016/j.tins.2015.02.001	216	21.60	4.68
8	Devaux Y. (2012). Clin. Chem.	10.1373/clinchem.2011.173823	209	16.08	4.46
9	Burney R. O. (2009). Mol. Hum. Reprod.	10.1093/molehr/gap068	205	12.81	1.42
10	Park C. K. (2014). Neuron	10.1016/j.neuron.2014.02.011	205	18.64	3.89

### 3.6 Analysis of organizations and institutions

We conducted a systematic analysis of contributions from various organizations or institutions. Among the top 10 most productive scientific institutions, Nanjing Medical University, Xuzhou Medical University, and Soochow University ranked in the top 3 (Table 6), and 80% of these top 10 institutions are in China, highlighting the dominant role of Chinese institutions in this research field. This promising result boosts the confidence of Chinese scientist in pain medicine and motivates them to intensify their efforts in studying the relationship between pain and miRNA.

**Table 6:** Analysis of organizations and institutions.

Rank	Organizations	Documents	Citations
1	Nanjing Medical University	37	784
2	Xuzhou Medical University	31	631
3	Soochow University	31	240
4	Shanghai Jiao Tong University	29	357
5	Huazhong University of Science and Technology	28	642
6	Southern Medical University	25	295
7	University of Carolina	23	449
8	Zhengzhou University	23	442
9	Hebrew University Jerusalem	22	1,221
10	Sun Yat-sen University	20	250

## 4 Discussion

We conducted a bibliometric analysis of scientific publications on the relationship between miRNA and neuropathic pain globally since 2000. Numerous articles have been published, and literature production kept a yearly growth, reflecting notable advances that have been made in understanding the relationship between miRNA and pain over the past two decades. Also, the establishment of extensive collaboration among different countries, and worldwide authors indicated an increase in global scientific concerns on the exploration of pain mechanisms.

The top three most frequently occurring keywords were “expression,” “neuropathic pain,” and “micro RNA,” which indicated that neuropathic pain was the most widely used disease model for the research of micro RNA and pain. The expression level of some microRNAs might be involved in the development and persistence of neuropathic pain. It’s widely known that miRNA plays a crucial role in post-transcriptional gene regulation and has demonstrated its potential value in pain progression and prognosis, including chronic, inflammatory, and neuropathic pain (Lopez-Gonzalez et al., 2017; Sakai and Suzuki, 2015).

Indeed, the revealed pain mechanisms have suggested a close relationship between miRNAs and the proteins or genes they regulate (Tao et al., 2018; Sakai and Suzuki, 2015; Jiangpan et al., 2016; Zhang et al., 2021; Hao et al., 2022; Zhang et al., 2020; Pan et al., 2018; Phạm et al., 2020; Favereaux et al., 2011; Tan et al., 2015). For instance, Zhang et al. (2021) discovered that miRNA-107 contributes to inflammatory pain by downregulating GLT-1 expression. Hao et al. (2022) discovered that the miRNA-22-Mtf1 signaling axis in the dorsal horn is critical for inflammatory pain progression. In neuropathic pain, which was the second most frequently occurring keyword, several novel miRNAs have been identified in recent years (Morchio et al., 2023). miRNA-103 was the first well-characterized miRNA in this field (Favereaux et al., 2011). Researchers discovered that subunits with Ca V 1.2 L-type calcium channels directly targeted miRNA-103. Calcium transient modulation was also observed in cultured spinal neurons, and altering the level of miRNA-103 expression altered pain behaviors (Favereaux et al., 2011). miRNA-128-3p was demonstrated to alleviate neuropathic pain by targeting ZEB1 through neuroinflammation inhibition (Zhang et al., 2020). MiRNA-155, which regulates inflammation-associated diseases, was upregulated, with its inhibition suppressing proinflammatory cytokines expression in the spinal cord of a CCI neuropathic pain rats model (Tan et al., 2015). miRNA-23a was identified to regulate neuropathic pain via TXNIP/NLRP3 inflammasome axis by directly targeting CXCR4 in a partial sciatic nerve ligation mouse model (Pan et al., 2018). Phạm et al. (2020) discovered that miRNA 146a-5p-encapsulated nanoparticles alleviate pain behaviors by inhibiting various inflammatory pathways in spinal microglia and reducing proinflammatory cytokine release. In the animal pain model, some experiments demonstrated that functional manipulation of miRNAs can suppress pain-related behavior in various pain diseases. Although these findings presented an extensive and dynamic change in microRNA expression in different pain models or animal studies, the strong relationship between pain development and miRNA changes was highlighted. This makes some promising miRNAs potential therapeutic molecular targets for pain medication for the high conservation of miRNAs and their target sequences among species (Sakai and Suzuki, 2015).

Similarly, researches were also conducted on humans suffering from pain disease and uncovered the biomarker potential of miRNA. Mari et al. (2019) found that miRNA-320 and miRNA-98 derived from circulating plasma were proven to successfully classify the pain type patients suffering in 70% of the cases. The circulating miRNA-320 was demonstrated to be responsible for post-traumatic pain by Linnstaedt et al. (2018). In our current visualized bibliometric study, further analysis of the most cited papers was performed, and we found that microRNA-21, miRNA-146a, miRNA-155, and miRNA-939 from peripheral were extensively studied in pain research for the critical role of inflammatory pain and neuropathic pain (Sommer et al., 2017; Ramanathan et al., 2019). Besides, spinal microglia-derived miRNA-124 and miRNA-155 were identified to serve critical roles in neuropathic pain (Tang et al., 2021). These promising microRNAs share the role of biomarkers in chronic pain in basic research. However, as for the application of miRNAs in clinical trials or pain management, it must be acknowledged that there is still a long way to go and some major obstacles and barriers remain before miRNA or its targeted gene is used as a medication, which the drug delivery strategy and the specificity of microRNA are the greatest barriers (Lopez-Gonzalez et al., 2017). To do so, the employment of viral vectors, as well as the incorporation of cholesterol molecules into a miRNA depressant or the sense strand of a miRNA mimic has been demonstrated to be efficacious strategies (Lopez-Gonzalez et al., 2017). Recently, packaging miRNA into extracellular vesicles, such as exosomes, proved to be a novel maneuver and could be potentially used as a candidate analgesic method (Zhang L. et al., 2023; Ramanathan et al., 2019; DaCunza et al., 2024; Kumar et al., 2024). Besides, more highly specified miRNAs and human tissue-derived miRNA data are warranted among the numerous candidates. Whatever, these studies, together with our current visualized analysis highlight the significance of miRNA in pain and show the direction of pain research in the future.

Our bibliometric analysis generated another interesting finding, which revealed that Chinese researchers contributed the most productions to the publications about pain and miRNA. Our study uncovered that 80% of the top 10 productive organizations and the top 3 most prolific scientists were from China. These findings were consistent with the comment in Nature, which noted that China has surpassed the United States in the total number of scientific publications, becoming the largest global producer of scientific articles (Tollefson, 2018). According to the recently announced Global Research Pulse report from Springer Nature (Springer Nature. 2024, August. China Impact Report), China is now the largest contributor to global research output. The production of scientific research is tightly related to the economic level of a country. China is world’s second-largest economy. In 2015, the Chinese government allocated about $400 billion to research and development, with this investment continuing to grow (Tollefson, 2018), among which, Natural Science Foundation of China (NSFC) provided great funding and basic research assistance from the national level. Significant national funding and support have encouraged a growing number of researchers to explore basic medicine and promote the achievement of international cooperation. A deep-going retrieve of the most productive author’s publications revealed that the top 1 ranked author Y. Zhang and his team established extensive collaboration with several internationally renowned hospitals or institutes, such as the East Tennessee State University, Henry Ford Hospital, and the Department of Physics Oakland University (Jia et al., 2018; Qiu et al., 2015). Extensive international exchanges and cooperations and the collaborative and open research environment would promote the launch of more innovative and comprehensive global research.

### 4.1 Limitations

Meanwhile, it is necessary to acknowledge the limitations of our bibliometric study. First, it included only English-language publications retrieved from WOSCC, excluding documents from other databases written in non-English languages. Second, the literature search did not entirely cover 2023, resulting in a decline in publications and a relatively unclear depiction of the overall trend. Third, we used the subject phrase “pain” in the data retrieval, which may have excluded papers with titles including “hurt” or “injury.” These limitations highlight the need for further research.

## 5 Conclusion

Our bibliometric study utilizes multiple visualized tools, including R language, bibliometric website, VOS viewer, and CiteSpace software, and fully uncovered the global tendency of research on the relationship between miRNA and pain by analyzing the number of publications, keywords, author data, countries, institutions, collaborations, citations, etc. The number of publications kept a steady growth, reflecting an increasing interest in and exploring the relationship between miRNAs and pain. Keywords analysis indicates that “expression,” “neuropathic pain,” and “microRNA” were the most frequently occurring words in this research field. Authors from China contributed to most publications. Among them, Yi Zhang was the most productive researcher. However, papers from prestigious journals were sparsely searched. More robust and globally recognized basic studies and clinical trials from renowned journals are demanded.

## Data Availability

The original contributions presented in the study are included in the article/supplementary material, further inquiries can be directed to the corresponding authors.

## Footnotes

^1^ http://bibliometric.com
